# Immunoglobulin subtype-coated bacteria are correlated with the disease activity of inflammatory bowel disease

**DOI:** 10.1038/s41598-021-96289-5

**Published:** 2021-08-17

**Authors:** Yutaro Masu, Yoshitake Kanazawa, Yoichi Kakuta, Yusuke Shimoyama, Motoyuki Onodera, Takeo Naito, Rintaro Moroi, Masatake Kuroha, Tomoya Kimura, Hisashi Shiga, Yoshitaka Kinouchi, Atsushi Masamune

**Affiliations:** 1 Division of Gastroenterology, Tohoku University Graduate School of Medicine, 1-1 Seiryo, Aoba, Sendai, 980-8574 Japan; 2 Department of Gastroenterology, South Miyagi Medical Center, Ogawara, Japan; 3 Health Administration Center, Center for the Advancement of Higher Education, Tohoku University, Sendai, Japan

**Keywords:** Inflammatory bowel disease, Clinical microbiology, Antimicrobial responses

## Abstract

Immune response involving various immunoglobulin (Ig) isotypes and subtypes to microbiome is involved in the pathogenesis and disease activity of inflammatory bowel diseases (IBDs). To clarify the presence of Ig-coated bacteria in the intestine and its association with disease activity in ulcerative colitis (UC) and Crohn’s disease (CD), we extracted and classified Ig-coated bacteria from fecal samples of 42 patients with IBD and 12 healthy controls (HCs) using flow cytometry and 16S ribosomal RNA sequence analysis. The percentage of bacteria coated with IgA and IgM was higher in patients with IBD than in HCs, and IgG-coated bacteria were found only in patients with IBD. Moreover, the percentages of bacteria coated with IgG1, IgG2, IgG3, and IgM in UC samples and IgG3, IgG4, and IgM in CD samples were correlated with disease activities. The proportions of *Bacteroides ovatus* and *Streptococcus* increased during the active phase of CD. Hence, the detailed analysis of Ig-coated bacteria and Ig subtypes using flow cytometry could aid in developing useful indicators of disease activity and identifying more disease-related bacteria, which could become novel treatment targets for IBDs.

## Introduction

Inflammatory bowel diseases (IBDs) such as ulcerative colitis (UC) and Crohn’s disease (CD) are chronic and relapsing inflammatory disorders of the gastrointestinal tract^1,2^. The pathogenesis of IBD is correlated with genetic variations, food, smoking, and dysregulated mucosal immune responses to commensal microbiota^3–5^. Although genetic factors were found to be associated with the onset of IBD, environmental factors, particularly gut microbiota, have been shown to play an important role. Several studies have supported the association between gut microbiota and IBD. Interleukin (IL)-10 knockout mice and HLA-B27 transgenic mice do not develop colitis under germ-free conditions^6,7^. The dysfunction of nucleotide-binding oligomerization domain-containing 2 (NOD2), which is a well-known susceptible gene of CD, is involved in the recognition of bacteria-derived muramyl dipeptide.

Recently, 16S rRNA gene sequencing using next-generation sequencers facilitates a comprehensive metagenomic analysis of human microbiota, and metagenomic analysis of intestinal microbiota reveals dysbiosis and decreased percentage of specific bacterial species such as Firmicutes in the intestine of patients with IBD. Some studies have evaluated Ig-coated bacteria using flow cytometric sorting and 16S rRNA gene sequencing. Previous studies showed that the proportion of IgA- and IgG-coated bacteria in the intestine of patients with IBD is high^8,9^. IgA-coated bacteria, which were isolated from the feces of patients with IBD, induced colitis in germ-free mice^10^. Based on these data, it appears that bacteria that are heavily coated with Ig play an important role in inducing immunological response and inflammation in the intestine of patients with IBD.

Various Ig isotypes and subtypes such as IgA1, IgA2, IgG1, IgG2, IgG3, IgG4, and IgM are involved in the immune response to gut microbiota. Each Ig subtype produces a different immune response via its respective Fc receptors. For example, IgG1 and IgG3 elicit complement activation and signal transduction via the Fcγ receptors I and III (FcγRI/FcγRIII), whereas IgG2 and IgG4 have a low inflammatory capacity^11,12^. This suggests that inflammatory responses differ among Ig subtypes, which might be related to disease pathogenesis and/or activity.

From the above mentioned data, we hypothesized that Ig-coated bacteria, especially bacteria coated with specific Ig subtypes, can aid in characterizing the two major phenotypes of IBD and/or be associated with disease activity. Therefore, we analyzed the proportion of Ig subtype-coated bacteria in the feces of healthy controls (HCs) and patients with IBD via flow cytometric cell sorting (FACS) and 16S rRNA gene sequencing.

## Materials and methods

### Participants and selection criteria

This study used fecal samples collected from patients with histologically and endoscopically diagnosed with UC or CD at the Tohoku University Hospital between February 2019 and June 2020. We excluded patients who were receiving treatment with antibiotics within 4 weeks before the collection of fecal samples and those who had a small intestinal stoma. Further, 12 volunteers without any known disease were included as HCs. The background characteristics of the participants are shown in Table 1. CD and UC were diagnosed based on clinical symptoms and endoscopic, radiographic, and histological findings according to the conventional criteria proposed by the Japanese Ministry of Health, Labour and Welfare^13^. The clinical score and laboratory tests were used to assess disease activity. The Clinical Activity Index (CAI) was used to evaluate the clinical score for UC and the Crohn’s Disease Activity Index (CDAI) for Crohn’s disease. Remission was defined as a CAI score < 5 and CDAI score < 150. The current study was conducted in accordance with the ethical standards of the Declaration of Helsinki and its later amendments along with the approval of the Ethics Committees of Tohoku University School of Medicine (approval number: 2018-1-602). Written informed consent was obtained from all participants.

**Table 1:** Characteristics of patients with IBD and healthy controls.

	HCs	UC patients	CD patients
Number of patients	12	20	22
Age^#^	35 ± 4.2	43 ± 17	42 ± 12*
Sex (male/female)	8/4	11/9	18/4
BMI^#^	22.9 ± 4.2	21.9 ± 4.2	20.8 ± 2.5
Disease activity (CAI/CDAI scores)^#^		4.5 ± 3.6	133.4 ± 87.2
Active/remission		9/11	8/14
Disease location n (%) (montreal classification)		E1: 2 (10)	L1: 3 (14)
		E2: 4 (20)	L2: 3 (14)
		E3: 14 (70)	L3: 15 (68)
**Current therapy n (%)**			
Azathioprine		5 (25)	4 (18)
Steroids		1 (5)	1 (4.5)
5-ASAs		17 (85)	11 (50)
Infliximab		8 (40)	2 (10)
Adalimumab		0 (0)	9 (41)
Ustekinumab		0 (0)	3 (14)

*HC* Healthy control, *UC* ulcerative colitis, *CD* Crohn’s disease, *SD* standard deviation, *CAI* Clinical Activity Index, *CDAI* Crohn’s disease activity.

*p < 0.05 (vs HCs), ^#^mean ± SD.

### Flow cytometry and sorting of Ig plus bacteria

Fecal samples were collected early in the morning using a feces sampling tool (AS ONE, Osaka, Japan) and stored in the Anaerobic Culture Kit Aneropack immediately after collection. Feces were processed within 12 h after collection. Approximately 100–500 mg of feces was placed in the Fast Prep Lysing Matrix D tube (MP Biomedicals, Illkirch, France) with 1 mL of phosphate-buffered saline (PBS). The samples were homogenized using FastPrep-24G (4.5 m/s, 5 s) and then centrifuged at 100 g for 15 min at 4 °C to remove large particles. The supernatants were filtered using 40-μm cell strainers, aliquoted into 100 μL volumes, and placed into 1.5-mL tubes. Each aliquot was washed with 1 mL of bacteria-staining buffer containing 1% (w/v) of bovine serum albumin in PBS and was centrifuged at 9000*g* for 5 min at 4 °C. A part of the aliquots was stored at − 80 °C for the measurement of Ig concentration in feces. After centrifugation, the bacterial pellets were resuspended in 100 μL of staining buffer containing 2 μL of biotinylated first antibody and were incubated for 30 min on ice. Parts of the bacterial pellets were dispensed for FACS analysis and were processed to second antibody staining. Other parts were processed to magnetic-activated cell sorting (MACS) before procession to the second antibody. Then, the latter fecal bacteria were washed with MACS buffer twice, incubated in MACS buffer containing 20 μL of anti-biotin MACS beads (Militenyi Biotec, Bergisch Gladbach, Germany) for 15 min on ice, and then sorted with MACS column according to the manufacturer’s protocol. Both the former and the latter samples processed via MACS separation were resuspended in 50 μL of blocking buffer containing 20% normal goat serum in bacteria-staining buffer, incubated for 20 min on ice, stained with AF647-conjugated second antibody for 20 min on ice, and then washed twice. To distinguish dead bacteria, the samples were stained with thiazole orange/propidium iodide, incubated for 5 min, washed twice, and then filtered using a 35-μm cell strainer. The stained bacterial samples were analyzed with FACS Aria III flow cytometer (Supplementary Fig. S1). For Ig-positive bacteria, up to ~ 1.0 × 10^6^ of samples were collected. The sorted bacteria were centrifuged at 12,000*g* for 10 min at 4 °C, and the pellets were stored at − 80 °C until the procedures for bacterial DNA extraction.

### DNA extraction

Bacterial DNA extraction was performed using the FastDNA SPIN Kit (MP Biomedical) according to the manufacturer’s protocol. In brief, the stored samples were suspended in 200 μL of DNase-free water and were placed in the Fast Prep Lysing Matrix tubes with 1 mL of cell lysis solution (CLS-TC). The samples were homogenized using FastPrep-24G (6 m/s, 40 s) and centrifuged at 14,000*g* for 10 min at 4 °C. The supernatants were collected to new tubes, added with 800 μL of SWS-M, incubated for 5 min, transferred to spin filter columns, and then centrifuged at 14,000*g* for 1 min at 20 °C. The samples were washed, added with 100 μL of DNase-free water, incubated for 5 min at 55 °C, and centrifuged at 14,000*g* for 1 min at 20 °C. The flow-through containing bacterial DNA was collected. The bacterial DNA extracts were treated with the PCR Inhibitor Removal Kit (ZYMORESWARCH, Irvine, CA) for additional purification.

### Bacterial 16S rRNA gene analysis

Amplification was carried out with the 2 × KAPA HiFi HotStart Ready Mix (Roche, Basel, Switzerland) using the purified bacterial DNA as a template and a set of universal primers targeting the V3–V4 region as amplification primers. Template DNA and 5 μL of 2 nM primers were added to the 1st PCR reaction mixture according to the manufacturer’s protocol. The conditions of the 1st PCR were as follows: 95 °C for 30 s, 72 °C for 30 s, 72 °C for 40 s, and 40 cycles. After the 1st PCR, the amplicons were cleaned up with the Agencourt AMPure XP (BECKMAN COULTER, Brea, CA). For the 2nd PCR, the Nextera XT Index Kit v2 Set A (Illumine, San Diego, CA) was used as an index primer. The conditions of the 2nd PCR were as follows: 95 °C for 30 s, 55 °C for 30 s, 72 °C for 40 s, and 8 cycles. After the 2nd PCR, the amplicons were cleaned up, as previously mentioned above. The base-pair length and the concentration of each amplicon were measured using the Agilent 2100 bioanalyzer (Agilent Technologies, Santa Clara, USA). The amplicons adjusted to 4 nM were combined into a library, added with 0.2 M NaOH, and then adjusted to 8 pM final DNA library. The final library was mixed with the PhiX control library (Illumina) adjusted to 8 pM, incubated at 96 °C for 2 min, and then incubated at 0 °C for 5 min. The mixed library was processed with the Illumina MiSeq platform (Illumina).

### Measurement of fecal free Ig concentrations

The fecal IgA, IgG1, IgG2, IgG3, IgG4, and IgM concentrations were measured using the LEGENDplex Human Immunoglobulin Isotyping Panel (6-plex) with V-bottom Plate (BioLegend) according to the manufacturer’s protocol, and the FCS file was analyzed using the LEGENDplex data analysis software. The fecal Ig concentrations were adjusted based on the amount of collected fecal samples.

### Statistical analysis

The percentage of Ig-coated bacteria and fecal free Ig concentrations were expressed as mean ± standard deviation (SD) in Supplementary Tables S1 and S2. The proportion of Ig-coated bacteria were analyzed using the Wilcoxon rank-sum test. Fecal free Ig concentrations were analyzed using the student’s *t*-test. The Fisher’s exact test was used to compare bacteria species and genera in whole feces and Ig-coated bacteria. Statistical calculations were performed with JMP version14 (SAS institute Inc., Cary, NC). A *p* value of < 0.05 was considered statistically significant. For diversity analysis of bacterial flora, the Shannon’s index was calculated as alpha diversity estimation, and the principal coordinate analysis (PCoA) based on unweighted UniFrac distance was calculated as beta diversity estimation using the QIIME2 software^14^. The diagnostic performance of Ig-coated bacteria and fecal free Ig concentration was evaluated using the receiver operating characteristic (ROC) curve and area under the curve (AUC).

## Results

### Demographic and clinical characteristics of the study cohort

A total of 54 fecal samples (20 UC, 22 CD, and 12 HC) were analyzed in this study (Table 1). The average age of patients with CD was significantly higher than that of HCs. Among patients with UC, 9 (45%) had active disease and 8 (40%) were treated with infliximab. Among those with CD, 8 (36%) had active disease and 14 (64%) were treated with biologics (infliximab, adalimumab, or ustekinumab).

### Ig-coated bacteria in patients with IBD

**Figure 1:**
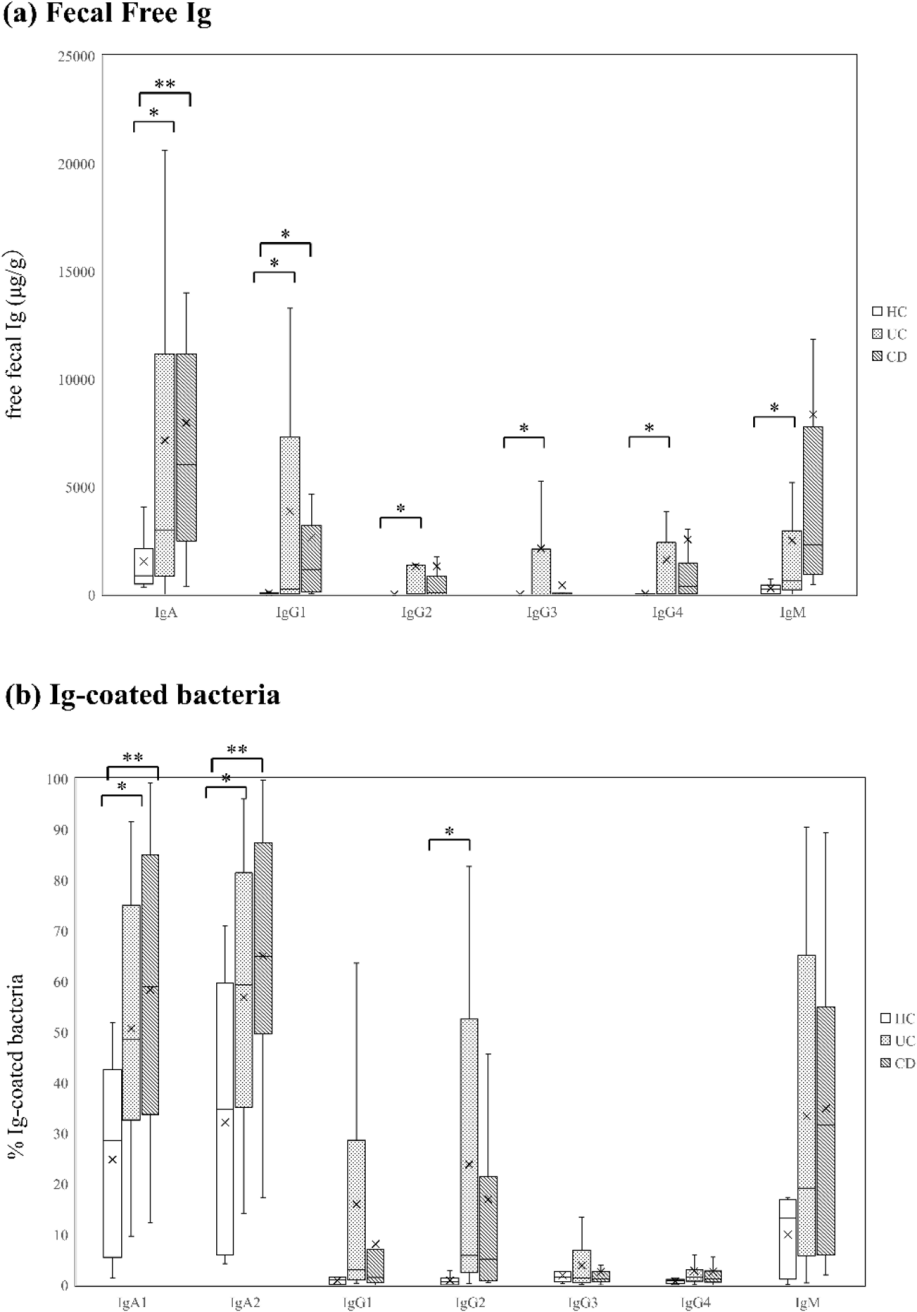
Free Ig and Ig-coated bacteria in fecal samples obtained from patients with IBD and HCs. (**a**) Box plot showing the concentration of fecal free Ig from HCs (n = 12), UC patients (n = 20), and CD patients (n = 22). The median (the bars in the boxes) concentrations of fecal free Ig are shown. (**b**) Box plot showing the flow cytometry analysis results for the ratio of each Ig-coated bacteria from HCs (n = 11), UC patients (n = 19), and CD patients (n = 21). The median (the bars in the boxes) concentrations of fecal free Ig are shown. The brackets indicate significant differences (Wilcoxon rank-sum test. *p*-values are shown *< 0.05, **< 0.005. The student’s *t*-test. *p*-values are shown ^†^< 0.05, ^††^< 0.005). *HC* Healthy control, *UC* ulcerative colitis, *CD* Crohn’s disease.

The UC samples had significantly higher free IgA, IgG1, IgG2, IgG3, IgG4, and IgM concentrations than the HC samples (*p* = 0.025, 0.032, 0.046, 0.042, 0.036, and 0.021, respectively); the CD samples had significantly higher IgA and IgG1 concentrations than the HC samples (*p* = 0.0032 and 0.039, respectively) (Fig. 1a and Supplementary Table S1). IgG2, IgG3, and IgG4 were rarely observed in the HC samples. In the analyses of percentages of Ig subtype-coated bacteria, the UC samples had a higher percentage of IgA1-, IgA2-, IgG1-, and IgG2-coated bacteria than the HC samples (*p* = 0.025, 0.0495, 0.022, and 0.0031, respectively) (Fig. 1b and Supplementary Table S2). Meanwhile, the percentage of IgA1-, IgA2-, and IgM-coated bacteria was higher in the CD samples than in the HC samples (*p* = 0.012, 0.16, and 0.00497, respectively). The proportion of IgG3- and IgG4-coated bacteria was extremely low and was only found in IBD samples.

### Correlation between Ig-coated bacteria and disease activity

**Figure 2:**
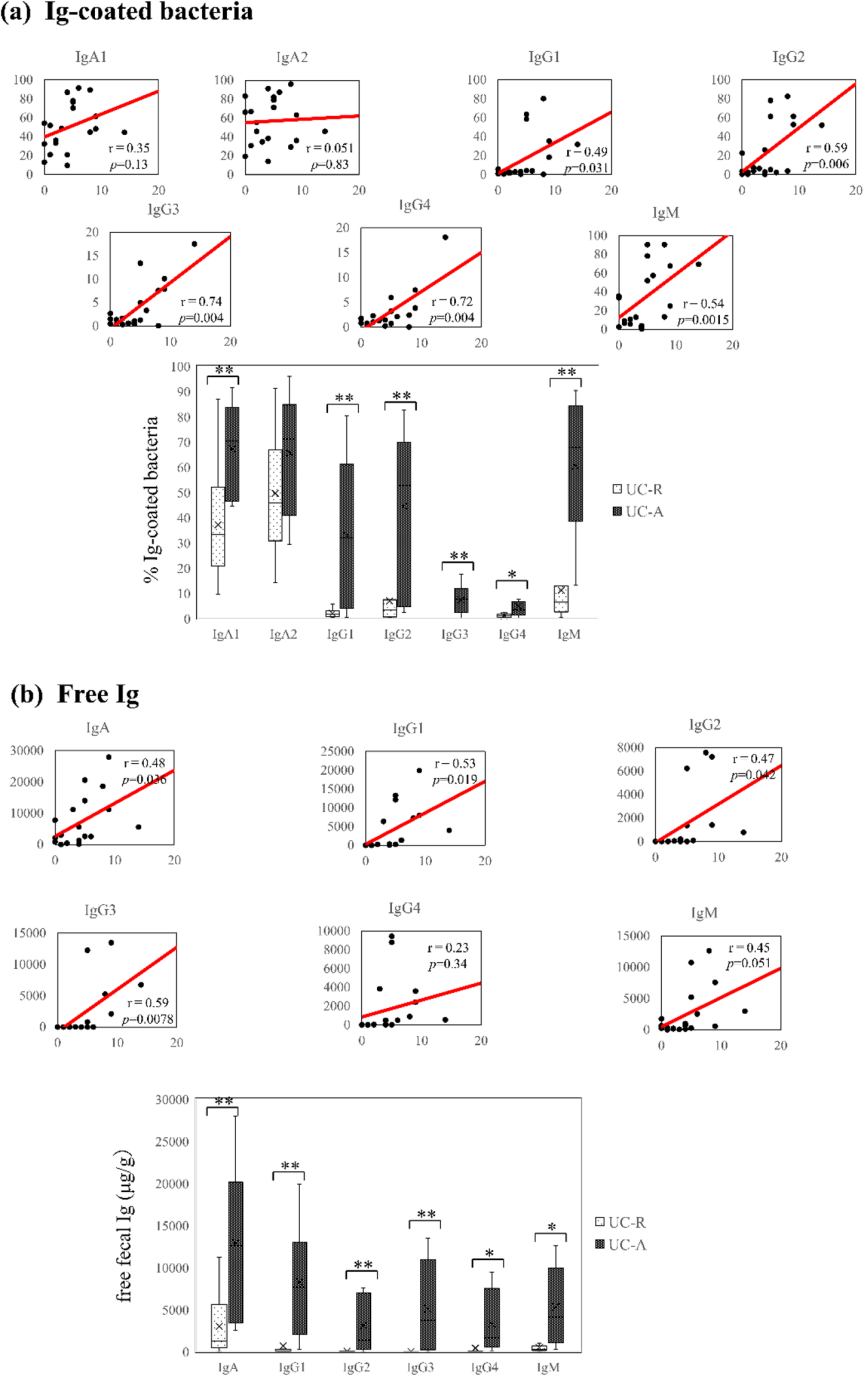
Correlation between UC activity as well as Ig-coated bacteria and fecal free Ig concentration. (**a**) The correlation between the ratio of each Ig-coated bacteria and the CAI score is shown in the graph. The CAI score was obtained at the time of fecal sample collection (n = 20). (**b**) Box plot showing the flow cytometry analysis results for the ratio of each Ig-coated bacteria in the UC-A (n = 9) and UC-R (n = 11) samples. UC-A had a more significant correlation with the ratio of each Ig-coated bacteria than UC-R. Disease activity was defined based on CAI. (**c**) The correlation between the concentration of fecal free Ig and the CAI score is shown in the graph. (**d**) Box plot showing the concentration of fecal free Ig in the UC-A (n = 8) and UC-R (n = 11) samples. UC-A was more correlated with the concentration of each fecal free Ig than UC-R. Spearman’s correlation analysis was performed, and the straight line in graphs (**a**) and (**c**) showed the regression line. The brackets in graphs (**b**) and (**d**) indicate significant differences. Wilcoxon rank-sum test, *p*-values are shown *< 0.05, **< 0.005. Student’s *t*-test. *p*-values are shown ^†^< 0.05, ^††^< 0.005). *UC* Ulcerative colitis, *CAI* Clinical Activity Index, *UC-A* ulcerative colitis in the active phase, *UC-R* ulcerative colitis in the active phase.

The proportions of all Ig subtype-coated bacteria, except the proportion of IgA2-coated bacteria, were significantly higher in the active-phase (UC-A) group than in the remission-phase (UC-R) group (e.g., IgM 60.4 ± 26.9 vs 11.2 ± 12.2, *p* = 0.00006, respectively) (Fig. 2a and Supplementary Table S3a). The fecal free Ig concentrations showed similar results (Fig. 2b and Supplementary Table S3b). The CAI score was correlated with the percentages of all IgG subtype- (IgG1–4) and IgM-coated bacteria (r = 0.49, 0.59, 0.74, 0.72, and 0.54; *p* = 0.031, 0.006, 0.004, 0.004, and 00,015, respectively), but it was not correlated with the percentages of IgA1 and IgA2-coated bacteria. Correlations were observed between the proportions of Ig (IgG1, IgG2, IgG3, and IgM)-coated bacteria and free Ig concentration in the fecal samples obtained from patients with UC (Supplementary Fig. S2).

**Figure 3:**
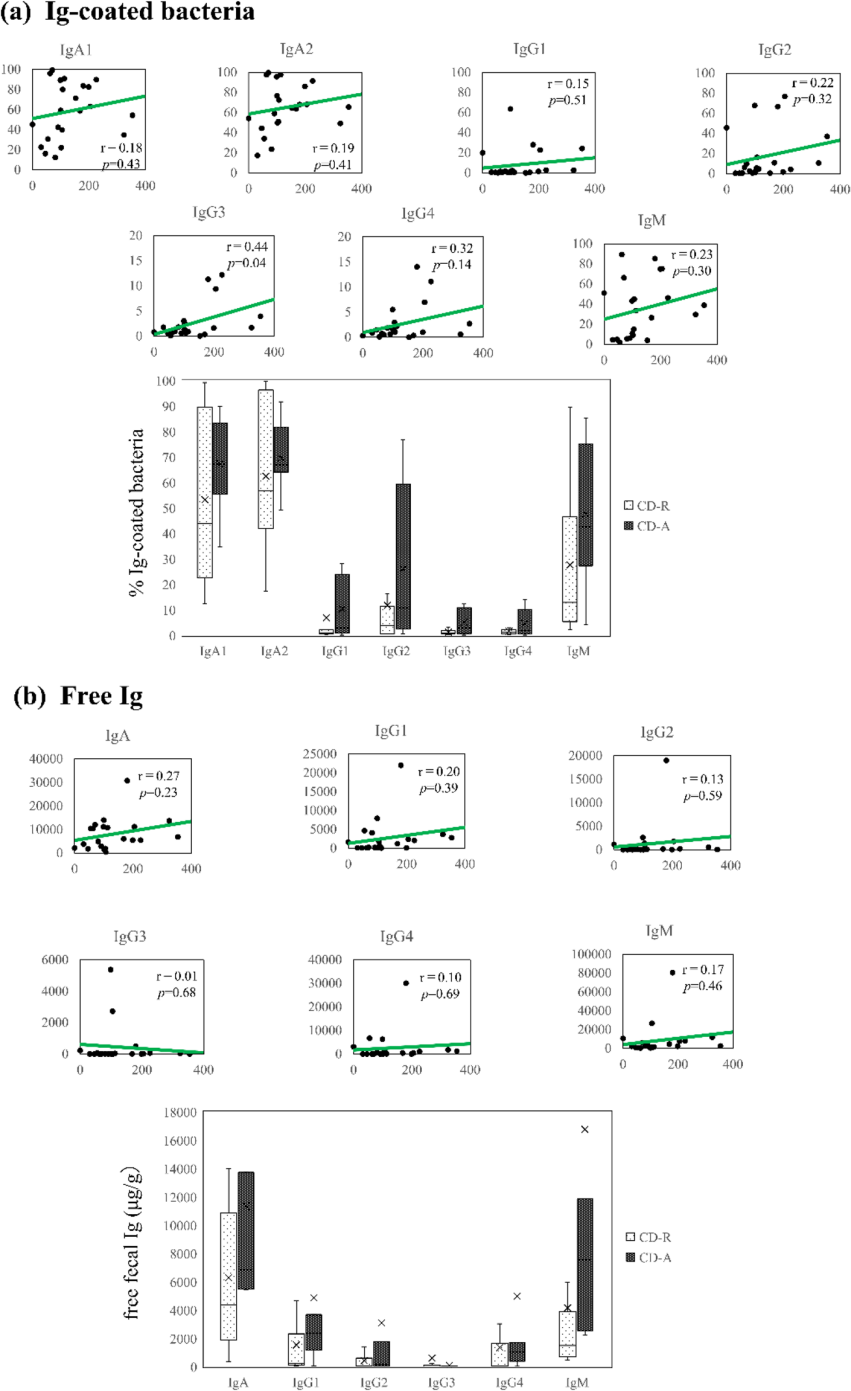
Correlation between CD activity as well as Ig-coated bacteria and fecal free Ig concentration. (**a**) The correlation between the ratio of each Ig-coated bacteria and the CDAI score is shown in the graph. The CDAI score was obtained at the time of fecal sample collection (n = 22). (**b**) Box plot showing the flow cytometry analysis results for the ratio of each Ig-coated bacteria in the CD-A (n = 8) and CD-R (n = 14) samples. CD-A was more correlated with the ratio of each Ig-coated bacteria than CD-R. Disease activity was defined based on the CDAI score. (**c**) The CDAI score was correlated with the concentration of each fecal free Ig. (**d**) Box plot showing the concentration of each fecal free Ig in the CD-A (n = 8) and CD-R (n = 11) samples. Unlike CD-R, CD-A was not correlated with the concentration of each fecal free Ig. Spearman’s correlation analysis was performed, and the straight line in graphs (**a**) and (**c**) is the regression line. The brackets in graphs (**b**) and (**d**) indicate significant differences. Wilcoxon rank-sum test. *p*-values are shown *< 0.05, **< 0.005. Student’s *t*-test. *p*-values are shown ^†^< 0.05, ^††^< 0.005). *CD* Crohn’s disease, *CDAI* Crohn’s Disease Activity Index, *CD-A* Crohn’s disease in the active phase, *CD-R* Crohn’s disease in the active phase.

These associations were also investigated in CD, but no difference was noted between the active-phase (CD-A) and remission-phase (CD-R) groups in relation to any of the Ig subtype-coated bacteria (Fig. 3a and Supplementary Table S4a) or fecal free Ig concentrations (Fig. 3b and Supplementary Table S4b). A correlation was noted between the CDAI score and the percentages of IgG3-coated bacteria (r = 0.44, *p* = 0.04). We confirmed a correlation between the proportions of Ig (IgG1, IgG2, and IgG4)-coated bacteria and free Ig concentration in CD (Supplementary Fig. S3).

### Percentage of Ig-coated bacteria and free Ig concentration in feces as an indicator of disease activity in UC

The results of ROC curve analysis to assess whether Ig-coated bacteria and free Ig concentration in feces can be used as indicators of disease activities are summarized in Table 2. In UC, each Ig-coated bacteria and fecal free Ig concentration showed higher AUCs. In particular, IgM-coated bacteria showed the highest AUC (0.96), and fecal free IgG1 showed the highest AUC (0.94). In CD, IgG3-coated bacteria showed the highest AUC (0.69), and fecal free IgM showed the highest AUC (0.82).

**Table 2:** Use of Ig-coated bacteria and free fecal Ig as a predictor of disease activities.

Disease		IgA1	IgA2	IgG1	IgG2	IgG3	IgG4	IgM
**(a) Ig-coated bacteria**								
UC	Sensitivity	1.00	0.56	0.89	0.67	0.78	0.78	1.00
	Specificity	0.64	0.63	0.91	1.00	1.00	0.91	0.82
	Cutoff (%)	45	71	3.84	52.2	3.34	2.31	13.3
	AUC	0.84	0.67	0.87	0.85	0.85	0.8	0.96
CD	Sensitivity	0.88	0.88	0.63	0.63	0.50	0.38	0.88
	Specificity	0.57	0.57	0.86	0.79	1.00	1.00	0.57
	Cut off (%)	54.3	63.9	2.69	10.6	3.93	7.00	26.5
	AUC	0.63	0.57	0.68	0.66	0.69	0.59	0.68

Disease		IgA	IgG1	IgG2	IgG3	IgG4	IgM	
**(b) Free fecal Ig**								
UC	Sensitivity	1.00	0.88	0.88	0.75	0.88	0.75	
	Specificity	0.64	0.91	0.91	1.00	0.91	1.00	
	Cutoff (μg/g)	2534	1345	82.5	802	496.9	2514	
	AUC	0.88	0.94	0.91	0.88	0.88	0.91	
CD	Sensitivity	1.00	0.71	0.71	1.00	0.86	0.86	
	Specificity	0.57	0.79	0.71	0.14	0.57	0.71	
	Cutoff (μg/g)	5459	2039	159.4	493.9	375.5	2552	
	AUC	0.72	0.69	0.66	0.43	0.66	0.82	

*UC* Ulcerative colitis, *CD* Crohn’s disease, *AUC* Area under the curve.

### Reduced diversity in fecal bacterial flora in IBD

**Figure 4:**
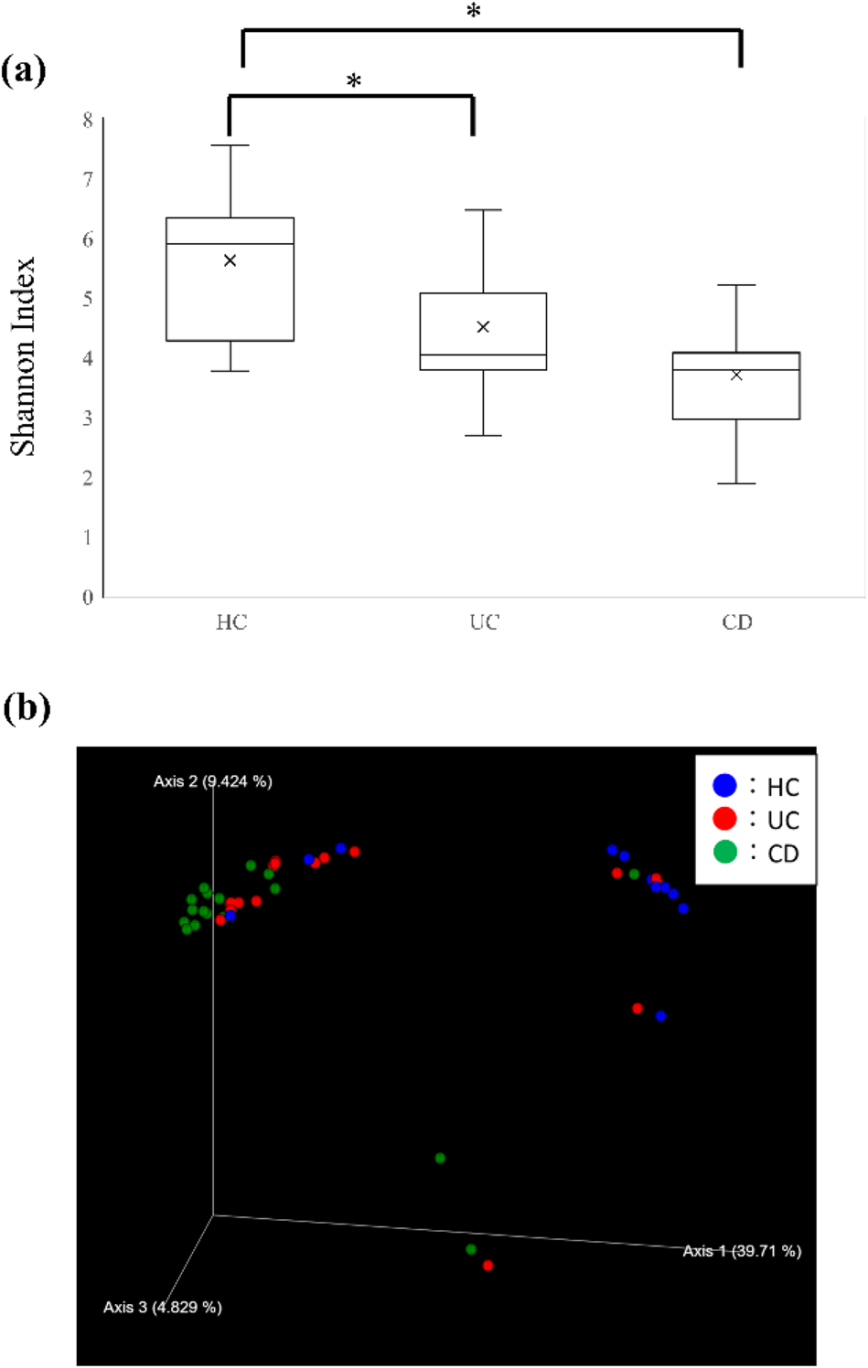
Diversities of fecal bacteria and Ig-coated bacteria. (**a**) Box plot showing α-diversity for the entire flora in HC and IBD samples. The brackets indicate significant differences (student’s *t*-test. *p*-values are shown *< 0.05, **< 0.005). (**b**) The HC and IBD samples are plotted in dot plots to show β-diversity. The blue, red, and green plots represent the HC, UC, and CD samples, respectively. Samples with similar flora structures are located close together, and samples with different flora structures are located far from each other. *HC* Healthy control, *UC* ulcerative colitis, *CD* Crohn’s disease.

We examined the diversity of the intestinal flora in the whole feces of HCs and patients with UC and CD (Fig. 4). Patients with UC and CD had decreased α-diversity than HCs (Shannon Index = 4.51 ± 1.11, 3.71 ± 1.33, and 5.64 ± 1.20, *p* = [UC vs HC] 0.212 and [CD vs HC] 0.000875, respectively). In a study of β-diversity, we confirmed the difference in bacterial composition in each disease group. HC and CD formed a cluster at a short distance. However, UC was dispersed in the cluster of HC and CD. The clusters of HC and CD were separated from each other, and the intestinal bacterial composition differed. By contrast, the population of UC did not have an evident cluster, and changes in gut flora composition varied.

### Higher percentage of *B. ovatus* and *Streptococcus* sp. in active CD and decreased percentage of several bacteria spp. in active UC

The detection rates of each bacterial species in the whole feces and Ig-coated bacteria were examined in the active and remission phases of both UC and CD (Table 3). At the species level, the percentage of *Bacteroides ovatus*, an IgA2-coated bacterium for CD, was significantly higher in the active phase than in the remission phase (*p* = 0.0047). The percentage of *Veillonella parvula*, an IgA2- and IgM-coated bacterium in UC, was significantly higher during the active phase than in the remission phase (*p* = 0.049 and 0.0087, respectively). Since the 16S rRNA universal primers (V3–V4 region) used in this study did not sufficiently identify bacteria at the species level, the genus level was also examined. The proportion of IgG2-coated bacteria from the genus *Streptococcus* was significantly higher in the active phase than in the remission phase (*p* = 0.048). In UC, we could not confirm the bacteria correlated to the active period. By contrast, a significantly lower proportion of several genera such as *Parabacteroides* (whole bacteria) (*p* = 0.018), *Blautia* (whole and IgA2-coated bacteria) (*p* = 0.018 and 0.049, respectively), *Megasphaera* (IgA1-coated bacteria) (*p* = 0.013), *Ralstonia* (IgA1-coated bacteria) (*p* = 0.013), *Ruminococcus* (whole and IgM-coated bacteria) (*p* = 0.0498 and 0.034, respectively), and *Roseburia* (whole and IgA1-, IgA2-, and IgM-coated bacteria) (*p* = 0.015, 0.0498, and 0.011, respectively) was detected in the active period.

**Table 3:** Bacterial species and genus whose detection rate changed according to disease activity.

	Disease	Sample type	Direction	*p* value
**(a) Species**				
*B. ovatus*	CD	IgA2	Increase	0.0047
		IgM		0.038
*V. parvula*	UC	IgA2	Decrease	0.049
		IgM		0.0087
**(b) Genera**				
*Streptococcus*	CD	IgG2	Increase	0.048
*Parabacteroides*	UC	Whole	Decrease	0.018
*Blautia*	UC	Whole		0.018
		IgA2		0.049
*Roseburia*	UC	Whole		0.015
		IgA2		0.0498
		IgM		0.011
*Ruminococcus*	UC	Whole		0.0498
		IgM		0.034
*Megasphaera*	UC	IgA1		0.013
*Ralstonia*	UC	IgA1		0.013

*UC* Ulcerative colitis, *CD* Crohn’s disease.

## Discussion

In this study, we investigated the response of each Ig to intestinal bacteria in IBD via FACS analysis. The major findings of this study are as follows. First, we confirmed that Ig coating of intestinal bacteria is enhanced in patients with IBD. Results showed increased IgA1 and IgA2 coating of bacteria in IBD. Ig-coated bacteria were observed to be coated with all IgG subtypes and IgM in UC patients and IgG1 in CD patients. Second, Ig (IgA1, IgA2, IgG1, IgG2, and IgM)-coated bacteria are correlated with disease activity in UC. Lastly, 16S rDNA analysis detected several bacterial species and genera that are associated with disease activity in UC and CD. These findings revealed enhanced production of Ig against pathogenic commensal bacteria in the gut, indicating that Ig’s these are involved in disease activity.

We confirmed increased bacterial coverage by various Ig isotypes in the fecal samples obtained from patients with IBD. Increased expression of TLR4, which recognizes the lipopolysaccharide of bacteria, has been confirmed in IBD, and abnormal immune responses to intestinal bacteria are believed to be associated with the pathology of IBD^15^. The increased coverages of intestinal bacteria with various Ig subtypes indicate that not only abnormalities in the natural immune system such as TLR signaling but also enhancement of acquired immunity in the intestinal tract are correlated with the pathophysiology of IBD.

The percentage of IgA1- and IgM-coated bacteria was higher in the active phase than in the remission phase of UC. By contrast, there was no difference in terms of the proportion of IgA- and IgM-coated bacteria between the active and remission phases of CD. Previous studies have reported that the percentages of IgA-coated bacteria and free IgA in feces are higher in IBD^9,16^. Palm et al. showed that IgA-coated bacteria can exacerbate enterocolitis^10^, which is considered a cause of IBD pathogenesis. IgA has two subtypes, which are as follows: IgA1 and IgA2. IgA2 is less susceptible to proteases than IgA1. Therefore, IgA2 is believed to be effective for the sequestration of intestinal bacteria. Furthermore, most IgA2 are present in the intestinal tract, particularly in the distal colon^17^. By contrast, the proportion of IgA1 in the large intestinal mucosa was higher in UC than in CD^18^. In this study, the rate of IgA1-coated bacteria was higher in active UC, which may reflect an increase in the number of IgA1 plasma cells in the submucosa. In addition, IgA1 accounts for 90% of IgA in the blood^19^, and active UC causes erosion and ulcers with bleeding in the mucous membrane. Therefore, serum IgA1 may affect the coverage of bacteria with IgA1. However, we could not distinguish mucosa-derived Ig from serum-derived Ig.

Although IgM bacterial coating is observed in infectious enterocolitis^20^ and is not an IBD-specific immune response, the levels of IgM-coated bacteria and free IgM in feces were significantly higher in active UC. Hence, it is considered a useful indicator of disease activity in intestinal inflammation. IgM is the first Ig to respond to antigen presentation and B-cell activation. Moreover, it appears in the early phase of the acquired immune response, and it can be a useful predictor of early inflammation and disease relapse. In our study, the proportion of IgM-coated bacteria was high in CD. However, no difference was observed between the remission and active phases. This may be attributed to the increase in the number of IgM-positive B cells during the remission phase of CD^21^. This result indicates that IgM-mediated enhancement of immune response may occur during the remission phase of CD.

In our results, the percentages of IgG-coated bacteria and the concentrations of free IgG in feces were higher in the active phase in UC and CD, particularly during the active phase of UC and IgG1 and IgG2 bacterial coatings were the main components. A correlation between UC activity and IgG3-coated bacteria was also observed. Previous studies have shown an increase in the proportion of IgG-producing plasma cells, especially IgG1-, IgG2-, and IgG3-producing plasma cells^22,23^, in the lamina propria of IBD patients compared with controls^8^, which is consistent with our results. IgG1 and IgG3 have greater proinflammatory effects than other IgG subtypes^24^. Further, correlations were also observed between fecal free Ig concentration and the percentages of IgG1-, IgG2- and IgG3-coated bacteria in UC. This result suggests that IgG produced in the mucosa could leak into the intestinal lumen and bind to bacteria. IBD inflammation may be partially caused by a defect in the intestinal mucosal barrier^25^. Hence, IgG-positive plasma cells are introduced in the lamina propria, and IgG produced from those plasma cells leak into the mucosa via erosive and/or leaky epithelia and contribute to the development of inflammation. By contrast, although CD has been found to increase the number of IgG1- and IgG2-positive cells in the lamina propria^8^, no significant difference was observed in the ratio of IgG1- and IgG2-coated bacteria between the active and remission phases. Unlike UC, inflammation is skipped in the entire gastrointestinal tract in CD and is away from the colon. We analyzed feces samples, which may not directly reflect local inflammation. Therefore, these results were influenced by differences in the location of inflammation between CD and UC.

The correlation between Ig-mediated immune response to intestinal bacteria and the pathophysiology and activity of IBD was confirmed. We analyzed the proportion of whole bacteria and Ig-coated bacteria via 16S rRNA gene sequencing. In the analysis using whole feces, we confirmed increased α-diversity in IBD fecal samples, and the β-diversity assessed with PCoA based on unweighted UniFrac showed an evident change in bacterial flora composition in the CD samples. This result was in accordance with that of previous reports showing that the diversity of intestinal bacteria is reduced in IBD^26–31^. In terms of β-diversity, the change in flora composition in UC was not as significant as that in CD. The flora composition during the remission phase in UC patients is similar to that in HCs^32^ Therefore, it was considered one of the reasons why UC did not differ from CD in this study.

Recently, several reports have identified disease-related and IBD activity-related bacteria from whole feces wherein a high number of bacterial species has been reported^33,34^. Ig-coated bacteria are already recognized by the immune system in the gut, and they are considered potentially pathogenic similar to IgA-coated bacteria^10^. Therefore, we investigated Ig-coated bacteria to identify the Ig-coated bacteria associated with disease activity. The ratio of *B. ovatus* and *Streptococcus* was high during the active phase of CD, and the proportion of bacterial species such as *Parabacteroides* and *Roseburia* was lower in the active phase of UC. *B. ovatus* and *Streptococcus* genera were found to be associated with disease activity, and they were observed during the active phase of CD. Recently, *B. ovatus* was found to significantly induce IgA production in the intestinal tract in mice^35^. *Streptococcus* spp. is also known as an oral bacterium that causes dental caries, and the proportion of this bacterium is high in the cultures of CD inflamed mucosal biopsy^36^. In this study, IgA2-coated bacteria were also detected in *B. ovatus* during the active phase. This finding is consistent with that of existing reports, and this indicates a relationship between *B. ovatus* and CD activity. In UC, we could not identify the bacterial species that increased in proportion during the active phase. However, the percentage of *V. parvula*, *Parabacteroides*, *Blautia*, *Roseburia*, *Ruminococcus*, *Megasphaera,* and *Ralstonia* decreased during the active period. Of these species, the proportion of species from the genus *Parabacteroides* was lower in the active phase than in the remission phase of UC^37^. In addition, the percentage of some species from the genus *Roseburia* decreased in UC^38^ and that the decrease in the proportion of some species from the genera *Blautia*, *Parabacteroides,* and *Roseburia* was associated with the development of ileal pouchitis after total colectomy^39,40^. In this study, the proportion of these bacterial species reduced in the active phase, which is in accordance with previous reports. Regarding the genus *Ruminococcus*, the proportion of *Ruminococcus bromii* was reduced in UC. Moreover, the transplantation of feces from donors rich in *Ruminococcus* is more effective than that from donors with limited bacteria in UC patients^41,42^. By contrast, *Ruminococcus gnavus* has been found to be associated with unsuccessful fecal transplantation^43^, and its effect on UC differs based on the species within the same genus. Our results are only representative of the comparison between fecal samples from different patients with active- or remission-phase disease, making it difficult to determine whether these specific bacteria are involved in the pathology of IBD or are affected as a result of IBD. However, previously reported findings suggest that these bacteria are associated with the pathology of IBD.

In this study, Ig-coated bacteria and fecal free Ig can be useful indicators of disease activity in UC. By contrast, there was a minimal change in the flora associated with disease activity in CD. Based on these results, in UC, immune response induced by intestinal bacteria is significantly involved in pathogenesis and disease activity. In CD, an abnormal immune response occurs even in the remission phase. Recently, the use of fecal microbiota transplantation (FMT) as a therapeutic option for refractory IBD has been investigated. Results showed that FMT has a therapeutic effect in UC. However, it does not have a sufficient therapeutic effect in CD^44^. Differences in the therapeutic effect of FMT in UC and CD may be correlated with such differences in the response to commensal microbiota in UC and CD.

The current study had several limitations, which are as follows: First, antibiotics were used in severe cases of CD. Hence, these cases were excluded from this study. Second, the effects of therapeutic agents for IBD, such as immunosuppressants and corticosteroids, were not evaluated. Third, bacterial 16S rRNA gene analysis was conducted using universal primers in a limited area. Thus, not all bacterial species were analyzed at the species level. Lastly, the number of participants was small to perform analyses stratified by several factors such as complications, disease activities, and therapeutic agents, which could result in changes in the gut flora. Additionally, there were several factors that could affect the results, such as the amount/balance of bacteria and antibodies, location of inflammation, sample collection method, and so on. To address these concerns, more data from patients with IBD, including those with a severe condition, should be collected in the future. Further investigation must be conducted to validate the pathophysiological role of Ig response and intestinal microbiota in the gut of IBD.

In conclusion, detailed Ig subtype analyses with FACS revealed that some Ig-coated bacteria and Ig subtypes can be useful indicators of disease activity in IBD, particularly UC. In addition, several bacterial species are correlated with disease activity in IBD. Although only a limited number of bacterial species were identified in this study, more disease-related bacteria could be identified by focusing on Ig-coated bacteria in combination with FACS. Further, novel therapies targeting specific bacteria could be considered in the future.

## Supplementary Information

Supplementary Information.
