# Supplementary material for: Immunoglobulin subtype-coated bacteria are correlated with the disease activity of inflammatory bowel disease
Source: Sci Rep. 2021 Aug 17;11:16672. doi: 10.1038/s41598-021-96289-5 (PMC8371132; doi:10.1038/s41598-021-96289-5)
Supplement: Supplementary file 1 — Supplementary Information. [file 41598_2021_96289_MOESM1_ESM.pdf]

Supplementary Materials

Immunoglobulin-mediated response and bacterial species analysis in inflammatory bowel disease

Supplementary Figure Legends

Figure S1. Identification and extraction of Ig-coated bacteria using a flow cytometer

The sequence of procedures for the identification of Ig-coated bacteria using FACSAriaIII. (a) Bacteria stained with each Ig antibody and TO/PI were gated with SSC and FSC. (b) Gated with FSC-area and FSC-height to remove doublets from bacteria. (c) Extracted live bacterial fractions that are both TO-positive and PI-negative. (d) After confirming the AF-647-positive fraction, which is an Ig-coated bacterial fraction, the Ig-coated bacteria fraction were classified. (e) The Ig-coated bacteria were reconfirmed via FACS

SSC: Side scatter, FSC: Forward scatter, TO: Thiazole orange, PI: Propidum iodide

Figure S2. Comparison of Ig-coated bacteria and fecal free Ig concentration in UC

To investigate the effect of fecal free Ig concentration on the rate of Ig-coated bacteria in UC, the correlation between fecal free Ig concentration (μg/g) and Ig bacterial coverage (%) was examined. A Spearman’s correlation analysis was performed, and the straight line in the graph indicated the regression line.

Figure S3. Comparison of Ig-coated bacteria and fecal free Ig concentration in CD

To investigate the effect of fecal free Ig concentration on the rate of Ig-coated bacteria in CD, the correlation between fecal free Ig concentration (μg/g) and Ig bacterial coverage (%) was examined. A Spearman’s correlation analysis was performed, and the straight line in the graph indicated the regression line.

Figure S1

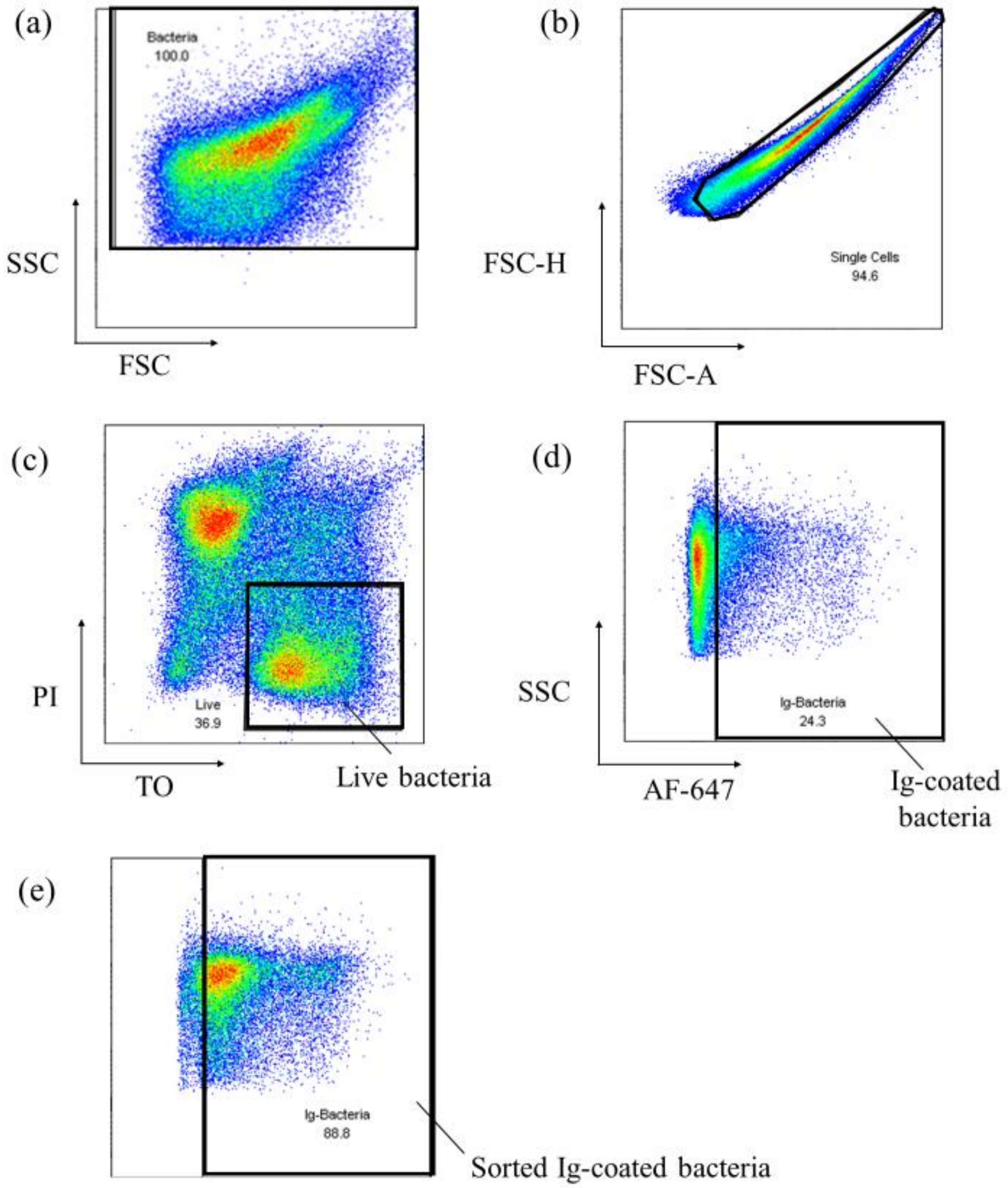

(a)
Bacteria
100.0
SSC
FSC
(b)
FSC-H
Single Cells
94.6
FSC-A
(c)
PI
Live
36.9
TO
Live bacteria
(d)
SSC
Ig-Bacteria
24.3
AF-647
Ig-coated bacteria
(e)
Ig-Bacteria
88.8
Sorted Ig-coated bacteria

Figure S2

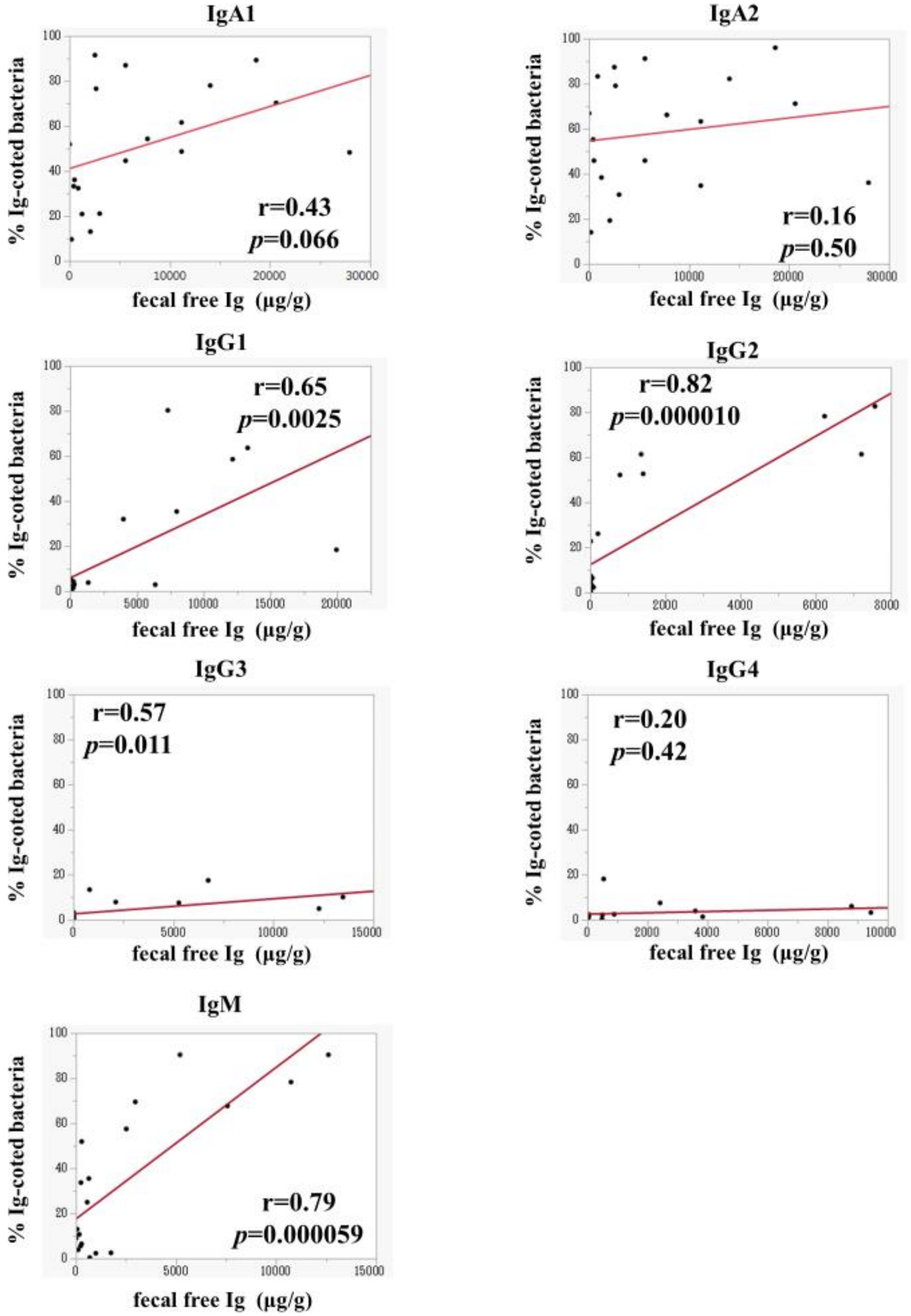

IgA1
% Ig-coted bacteria
fecal free Ig (μg/g)
r=0.43
p=0.066
IgA2
% Ig-coted bacteria
fecal free Ig (μg/g)
r=0.16
p=0.50
IgG1
% Ig-coted bacteria
fecal free Ig (μg/g)
r=0.65
p=0.0025
IgG2
% Ig-coted bacteria
fecal free Ig (μg/g)
r=0.82
p=0.000010
IgG3
% Ig-coted bacteria
fecal free Ig (μg/g)
r=0.57
p=0.011
IgG4
% Ig-coted bacteria
fecal free Ig (μg/g)
r=0.20
p=0.42
IgM
% Ig-coted bacteria
fecal free Ig (μg/g)
r=0.79
p=0.000059

Figure S3

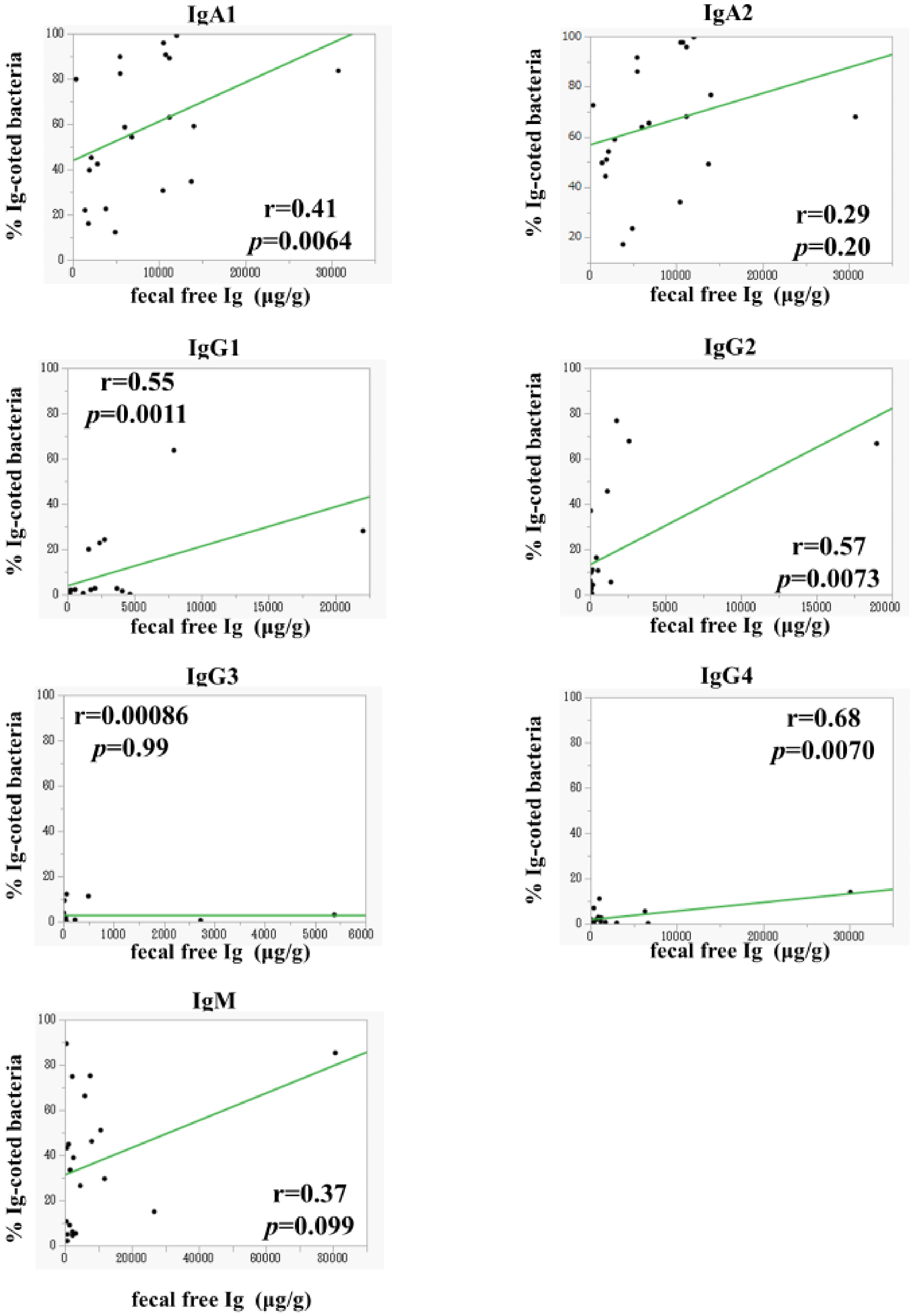
IgA1
% Ig-coted bacteria
fecal free Ig (μg/g)
r=0.41
p=0.0064
0
10000
20000
30000
0
20
40
60
80
100
IgA2
% Ig-coted bacteria
fecal free Ig (μg/g)
r=0.29
p=0.20
0
10000
20000
30000
20
40
60
80
100
IgG1
% Ig-coted bacteria
fecal free Ig (μg/g)
r=0.55
p=0.0011
0
5000
10000
15000
20000
0
20
40
60
80
100
IgG2
% Ig-coted bacteria
fecal free Ig (μg/g)
r=0.57
p=0.0073
0
5000
10000
15000
20000
0
20
40
60
80
100
IgG3
% Ig-coted bacteria
fecal free Ig (μg/g)
r=0.00086
p=0.99
0
1000
2000
3000
4000
5000
6000
0
20
40
60
80
100
IgG4
% Ig-coted bacteria
fecal free Ig (μg/g)
r=0.68
p=0.0070
0
10000
20000
30000
0
20
40
60
80
100
IgM
% Ig-coted bacteria
fecal free Ig (μg/g)
r=0.37
p=0.099
0
20000
40000
60000
80000
0
20
40
60
80
100

Supplementary Table S1. Fecal free Ig concentration in HC and IBD samples

	HC samples	IBD samples		
IgA (μg/g)	1541 ± 1665	UC	7160 ± 8093	(p = 0.025)
		CD	7976 ± 6806	(p = 0.0032)
IgG1 (μg/g)	68 ± 109	UC	3876 ± 1665	(p = 0.032)
		CD	2651 ± 4881	(p = 0.039)
IgG2 (μg/g)	9 ± 16	UC	1315 ± 2580	(p = 0.046)
		CD	1313 ± 4109	(p = 0.28)
IgG3 (μg/g)	9 ± 22	UC	2141 ± 4238	(p = 0.042)
		CD	433.9 ± 1279	(p = 0.26)
IgG4 (μg/g)	34 ± 43	UC	1614 ± 2907	(p = 0.036)
		CD	2559 ± 6591	(p = 0.20)
IgM (μg/g)	290 ± 254	UC	2506 ± 3802	(p = 0.021)
		CD	8357 ± 17620	(p = 0.13)

※ mean ± standard deviation

HC: Healthy control, IBD: Inflammatory bowel disease

UC: Ulcerative colitis, CD: Crohn’s disease

Supplementary Table S2. Ratio of each Ig-coated bacteria in HC and IBD samples

	HC samples	IBD samples		
IgA1 (%)	27.4±18.9	UC	50.7 ± 25.3	(p = 0.025)
		CD	58.3 ± 28.1	(p = 0.012)
IgA2 (%)	33.7 ± 23.4	UC	56.8 ± 25.2	(p = 0.0495)
		CD	65.0 ± 23.8	(p = 0.016)
IgG1 (%)	0.7 ± 0.6	UC	16.0 ± 24.7	(p = 0.022)
		CD	8.1 ± 15.4	(p = 0.23)
IgG2 (%)	0.9 ± 0.9	UC	23.8 ± 29.1	(p = 0.0031)
		CD	16.9 ± 24.7	(p = 0.010)
IgG3 (%)	1.3 ± 1.6	UC	3.9 ± 4.9	(p = 0.76)
		CD	2.6 ± 3.6	(p = 0.65)
IgG4 (%)	0.6 ± 0.5	UC	2.8 ± 4.1	(p = 0.20)
		CD	2.7 ± 3.7	(p = 0.18)
IgM (%)	8.7 ± 6.8	UC	33.4 ± 31.9	(p = 0.069)
		CD	34.9 ± 29.0	(p = 0.00497)

※ mean ± SD (Standard Deviation)

HC: Healthy control, IBD: Inflammatory bowel disease

UC: Ulcerative colitis, CD: Crohn’s disease

Supplementary Table S3.

Ig-coated bacterial ratio and fecal free Ig concentration during the active/remission phase of UC

(a) Ig-coated bacterial ratio during the active/remission phase of UC

	UC-A samples	UC-R samples	*p* value
IgA1 (%)	67.2 ± 18.4	37.1 ± 22.4	0.012
IgA2 (%)	65.6 ± 23.6	49.7 ± 25.2	0.21
IgG1 (%)	33.0 ± 29.1	2.01 ± 1.55	0.0062
IgG2 (%)	44.5 ± 32.1	6.95 ± 8.98	0.0098
IgG3 (%)	7.35 ± 5.67	1.02 ± 0.721	0.0098
IgG4 (%)	4.89 ± 5.48	1.11 ± 0.667	0.028
IgM (%)	60.4 ± 26.9	11.2 ± 12.2	0.00006

(b) Fecal free Ig concentration during the active/remission phase of UC

	UC-A samples	UC-R samples	*p* value
IgA (μg/g)	12900 ± 9174	2986 ± 3671	0.0045
IgG1 (μg/g)	8278 ± 675.3	675.3 ± 1891	0.020
IgG2 (μg/g)	3080 ± 3311	30.65 ± 59.90	0.0067
IgG3 (μg/g)	5083 ± 5394	2.191 ± 2.260	0.0057
IgG4 (μg/g)	3274 ± 3795	407.1 ± 1147	0.029
IgM (μg/g)	5306 ± 4616	469.4 ± 523.0	0.0029

※ mean ± standard deviation

UC-A: Ulcerative colitis active phase, UC-R: Ulcerative colitis remission phase

Supplementary Table S4.

Ig-coated bacterial ratio and fecal free Ig concentration during the active/remission phase of CD

(a) Ig-coated bacterial ratio during the active/remission phase of CD

	CD-A samples	CD-R samples	*p* value
IgA1 (%)	67.2 ± 18.3	53.2 ± 31.9	0.36
IgA2 (%)	69.6 ± 13.4	62.4 ± 28.2	0.61
IgG1 (%)	10.3 ± 12.3	6.84 ± 17.1	0.18
IgG2 (%)	26.1 ± 30.6	11.7 ± 20.0	0.23
IgG3 (%)	5.06 ± 5.08	1.21 ± 0.825	0.16
IgG4 (%)	4.59 ± 5.45	1.55 ± 1.40	0.52
IgM (%)	1.55 ± 1.40	27.6 ± 27.6	0.18

(b) Fecal free Ig concentration during the active/remission phase of CD

	CD-A samples	CD-R samples	*p* value
IgA (μg/g)	11350 ± 9130	6290 ± 4858	0.11
IgG1 (μg/g)	4874 ± 7645	1540 ± 2384	0.14
IgG2 (μg/g)	3095 ± 7032	422.4 ± 766.1	0.17
IgG3 (μg/g)	91.21 ± 179.1	605.2 ± 1551	0.40
IgG4 (μg/g)	4974 ± 11086	1352 ± 2325	0.24
IgM (μg/g)	16770 ± 28340	4153 ± 7049	0.12

※ mean ± standard deviation

CD-A: Crohn’s disease active phase, CD-R: Crohn’s disease remission phase
